# Associations between TLR4 Polymorphisms and Open Angle Glaucoma: A Meta-Analysis

**DOI:** 10.1155/2019/6707650

**Published:** 2019-07-24

**Authors:** Zhongjing Lin, Shouyue Huang, Jun Sun, Bing Xie, Yisheng Zhong

**Affiliations:** Department of Ophthalmology, Ruijin Hospital Affiliated Medical School, Shanghai Jiaotong University, 197 Ruijin Er Road, 200025 Shanghai, China

## Abstract

**Background:**

Previous studies exploring the association between toll-like receptor 4 (TLR4) polymorphisms and open angle glaucoma (OAG) presented inconsistent results. We aimed to investigate the association between TLR4 polymorphisms and OAG.

**Methods:**

A systematic literature search was conducted in PubMed, EMBASE, ISI Web of Knowledge, and the Cochrane Library up to 31 December 2018. Odds ratio (OR) and 95% confidence interval (95%CI) were calculated, followed by stratification analyses according to ethnicity and glaucoma subtype.

**Results:**

TLR4 rs7037117 polymorphism had significant associations with increased risk of OAG in allelic model (OR=1.25; 95%CI: 1.09-1.44; P=0.002) and recessive model (OR=1.49; 95%CI: 1.08-2.04; P=0.01). With regard to rs10759930, rs12377632, and rs2149356, the results showed significant increased risks in all genetic models (all P<0.05), whereas, for rs1927914, rs11536889, and rs7045953, no significant associations were identified in any genetic model (all P>0.05). Furthermore, the association of rs1927911 with OAG risk was found to be significant in recessive model (OR=1.34; 95%CI: 1.06-1.71; P=0.02). As for rs4986790 and rs4986791, meta-analyses were not performed due to the limited number of studies and the ethnic differences. Subgroup analysis indicated that the above polymorphisms with significant differences might increase the susceptibility in POAG patients. As for the ethnicity, rs7037117, rs10759930, and rs1927911 might increase the risk in Asians, while rs12377632 and rs2149356 might increase the risk in Asians and Mexicans.

**Conclusion:**

The meta-analysis highlighted that certain mutations of some TLR4 polymorphisms might increase the susceptibility of OAG. However, TLR4 polymorphisms are still far from being candidate genetic biomarkers for OAG. Additional researches involving larger scale epidemiological studies are warranted to validate our results.

## 1. Introduction

Glaucoma is a group of neurodegenerative diseases that can lead to irreversible blindness. The total amount of glaucomatous patients aged 40-80 years was estimated to be 64.3 million in 2013, and it will dramatically increase to 111.8 million in 2040 [1]. Open angle glaucoma (OAG) is the most common form, accounting for about 85% of glaucoma cases. It is characterized by progressive degeneration of retinal ganglion cells (RGCs) and subsequent irrevocable vision loss, which can occur with either elevated intraocular pressure (IOP) or normal IOP, commonly termed as primary open-angle glaucoma (POAG) and normal tension glaucoma (NTG), respectively. The precise pathophysiology of OAG is complex and the mechanism remains obscure. Despite the major risk factor of increased IOP, genetic variations also play an important role in this disease profile [2]. Mutations in various genes have been reported in glaucoma patients from multiple populations [3–5]. Therefore, close attention has been paid to the association between single-nucleotide polymorphisms (SNPs) of genes and the development of OAG.

It has been reported that the immune regulation is involved in the pathogenesis of the degeneration of the RGCs in glaucoma [6, 7]. One of the key proteins in the immune response is toll-like receptors (TLRs) [8]. TLRs have been recognized as a family of pattern-recognition receptors and have a specific role in the human immune systems. TLR4, one of the most characterized members in the TLR family, has been reported to recognize lipopolysaccharides (LPS) and heat shock proteins (HSP), initiating immune responses that interact with exogenous and endogenous ligands. TLR4 has been demonstrated to affect the risk of various multifactorial disorders, such as retinal ischemia/reperfusion injury, glaucoma, and diabetic retinopathy [9]. In in vivo studies, the results suggested that the inhibition of TLR4 signaling might be a promising candidate for the treatment of glaucoma [10–12]. Moreover, prior investigations revealed an upregulation of TLRs in the glaucomatous human retina by proteomic and immunohistochemical analyses, and in vitro findings in retinal microglia and astrocytes cultures suggested a TLR-mediated response leading to increased cytokine production and antigen presentation, indicating that TLRs play a role modulating the immune response in glaucoma [13].

In the past decades, several case-control studies varying in design, ethnicity, and sample size are examining the potential association between TLR4 polymorphisms and glaucoma, only to find inconclusive or contradictory results [14–21]. For example, Takano et al. [16] concluded that TLR4 polymorphisms were associated with POAG and NTG patients in Japanese individuals. However, Suh et al. [15] demonstrated that TLR4 polymorphisms may not be an associated factor in NTG pathogenesis in the South Korean population. Mousa et al. [18] indicated that there were no obvious associations between TLR4 rs4986791 and POAG in a Saudi cohort. Therefore, we aimed to investigate the possible association between TLR4 polymorphisms and OAG using a meta-analysis approach, followed by stratification analyses according to ethnicity and the subtype of glaucoma.

## 2. Materials and Methods

### 2.1. Literature Search

The review searching process conformed to the Preferred Reporting Items for Systematic Reviews and Meta-Analyses (PRISMA) guidelines. We performed a literature search using PubMed, EMBASE, ISI Web of Knowledge, and the Cochrane Library without language or time restrictions. The final search was conducted on 31 December 2018. We searched the databases using the following key words in different combinations: toll-like receptor, polymorphism, and glaucoma. Furthermore, the reference lists of the relevant publications were also checked carefully to identify any additional study not found through electronic searching.

### 2.2. Inclusion and Exclusion Criteria

Eligible studies were selected if they were case-control studies which evaluated the association between TLR4 polymorphisms and OAG and presented the distributions of TLR4 alleles for both cases and controls. All animal studies, case reports, abstracts from conferences, studies with incomplete data, and reviews were excluded. Although we did not define language in the review process, the articles in the final analysis were all in English.

### 2.3. Data Extraction

Two review authors extracted all the required data independently from the included articles. The extracted information included the following: first author, publication year, country, number of cases and controls, and genotype frequencies for cases and controls. Divergences, if existed, would be eliminated by discussion.

### 2.4. Quality Assessment

The Newcastle-Ottawa Scale (NOS) was used for the quality assessment in our meta-analysis. This quality scoring system contains three broad perspectives, divided into 8 items specifically, and the total score ranges as zero up to nine stars [22]. A score of 6 or higher indicates that the study has adequate quality. Two review authors subjectively scored each involved study and any differences would be resolved by discussion if applicable.

### 2.5. Statistical Analysis

Statistical analysis was performed using Revman software (version 5.3; Cochrane Collaboration, Oxford, United Kingdom). If the Hardy-Weinberg Equilibrium (HWE) was not reported in the original papers, we would calculate HWE p-value for the control group of each study using the Chi-square test, and p-value < 0.05 was defined as a significant disequilibrium. We examined the heterogeneity among included studies using the Chi-squared test and I^2^ test. For a heterogeneity result of I^2^ > 50% the random effects model was implemented; otherwise the fixed effects model was used to calculate pooled odds ratio (OR) and 95% confidence interval (CI) values [23]. A p-value < 0.05 was considered significant for statistical analysis. The allelic model (2 vs. 1), dominant model (22+12 vs. 11), and recessive model (22 vs. 12+11) were calculated to evaluate the association between TLR4 polymorphisms and OAG [24]. In order to detect potential publication bias, Begg's and Egger's tests were calculated using Stata (version 14; StataCorp, College Station, Texas).

## 3. Results

**Figure 1:**
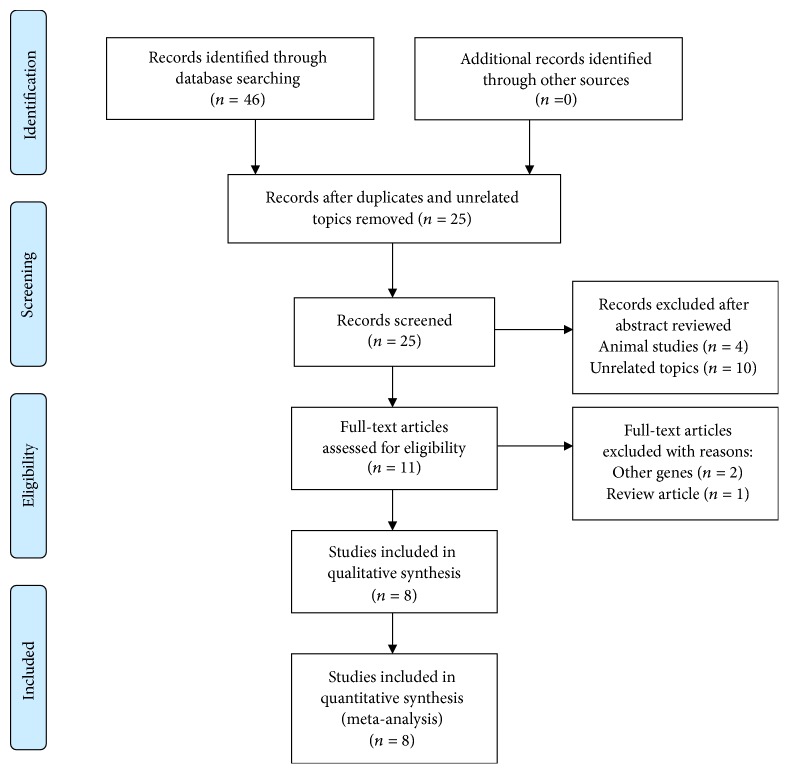
PRISMA flow diagram of the selection process for studies investigating the association between TLR 4 polymorphisms and open angle glaucoma.

According to the literature search strategy, eight studies were included in this meta-analysis (Figure 1). Relevant features of the included studies were consistent in the two review authors and the results were summarized in Table 1. Among the enrolled studies, Takano et al. [16] conducted a multicenter study about TLR4 genes and included four study groups, so we separately compared the two OAG patient groups (NTG group and POAG group) with the normal control group. The study populations came from different countries: Japan, Korea, China, Saudi Arabia, and Mexico. According to the NOS scoring system, the two reviewer authors rated the same scores and all the selected studies in our meta-analysis were considered to be of high quality. Furthermore, all the included studies have shown that genotype distributions of all SNPs in the control groups were consistent with HWE in the original papers.

**Figure 2:**
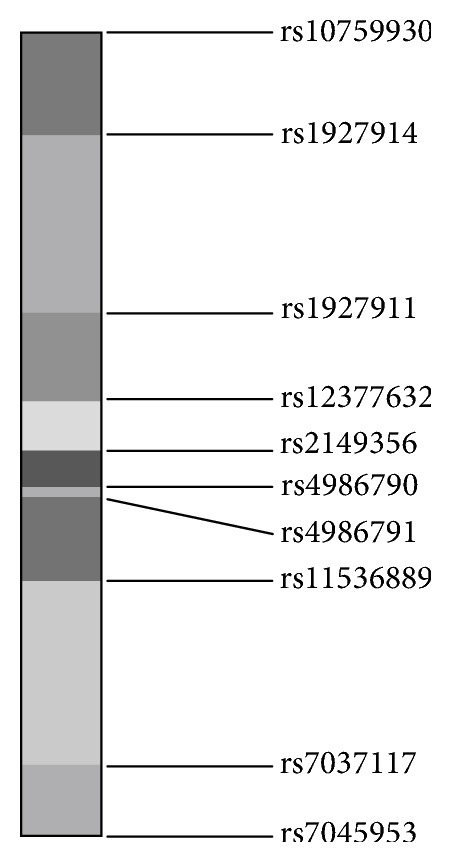
The genetic locations of 10 SNPs of the TLR4 gene. Two SNPs (rs10759930 and rs1927914) located on the 5' untranslated region. Three SNPs (rs1927911, rs12377632, and rs2149356) were in the intronic region and two SNPs (rs4986790, rs4986791) were in the nonsynonymous exonic region. Three SNPs (rs11536889, rs7037117, and rs7045953) located on the 3' untranslated region.

A total of 10 SNPs were involved in the included studies: rs7037117, rs10759930, rs1927914, rs1927911, rs12377632, rs2149356, rs11536889, rs7045953, rs4986790, and rs4986791. Among 10 SNPs on the TLR4 gene, two SNPs located on the 5' untranslated region (UTR) (rs10759930 and rs1927914), and three intronic SNPs (rs1927911, rs12377632, and rs2149356), two nonsynonymous exonic SNPs (rs4986790, rs4986791), and three SNPs located on the 3' UTR (rs11536889, rs7037117, and rs7045953). The locations of the above SNPs are shown in Figure 2. As for rs4986790 and rs4986791, only two research teams from Saudi Arabia and Mexico focused on these SNPs. Considering the ethnic differences and the limited number of studies, quantitative meta-analyses were not performed.

**Figure 3:**
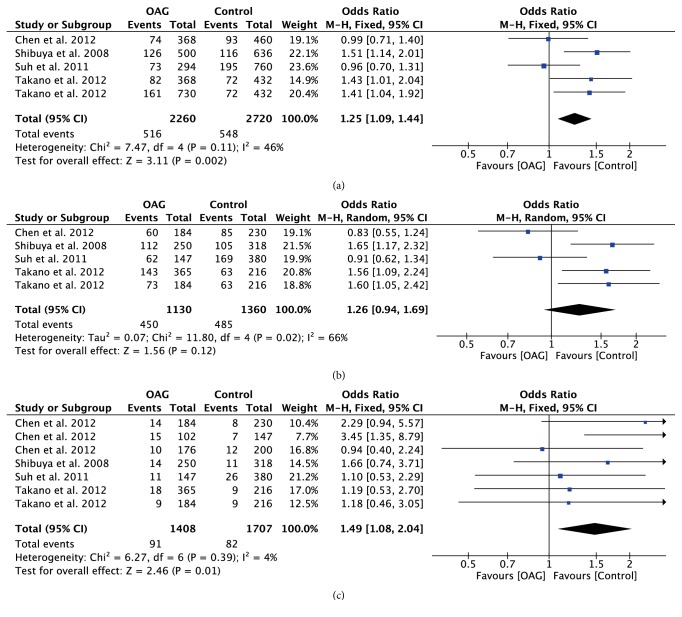
Forest plot of the association between TLR4 rs7037117 polymorphism and open angle glaucoma for all five models. (a) Allelic model, (b) dominant model, and (c) recessive model.

The association strengths between TLR polymorphisms and OAG are presented in Table 2. Stratification analyses were further performed based on ethnicity or glaucoma subtype. Combined data revealed that TLR4 rs7037117 polymorphism had significant associations with increased risk of OAG in allelic model (OR=1.25; 95%CI: 1.09-1.44; P=0.002) and recessive model (OR=1.49; 95%CI: 1.08-2.04; P=0.01) (Figure 3). Stratification analyses also suggested that the role of GG genotype increased the risk of OAG in Asian patients, especially in POAG patients (OR=1.71; 95%CI: 1.10-2.66; P=0.02).

**Figure 4:**
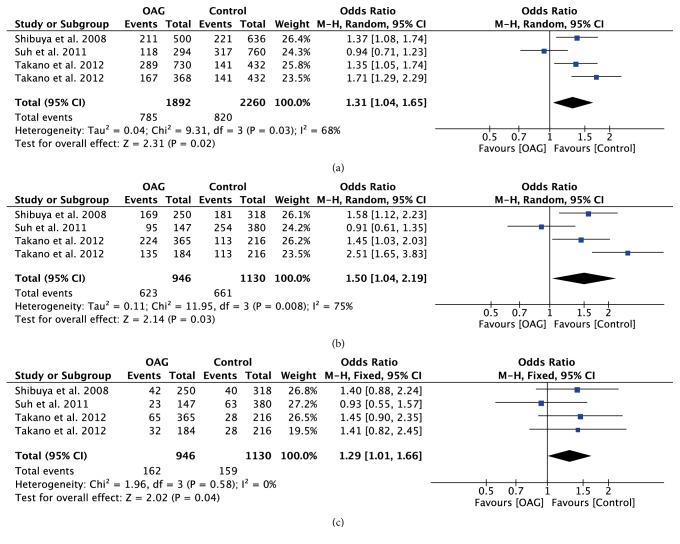
Forest plot of the association between TLR4 rs10759930 polymorphism and open angle glaucoma for all five models. (a) Allelic model, (b) dominant model, and (c) recessive model.

**Figure 5:**
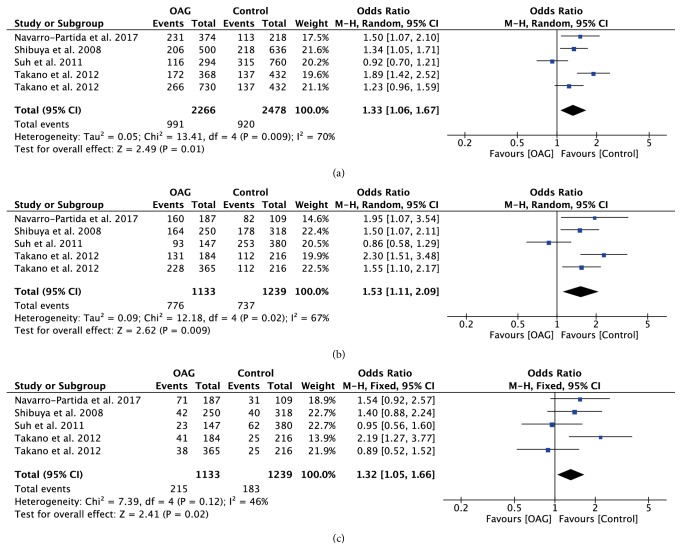
Forest plot of the association between TLR4 rs12377632 polymorphism and open angle glaucoma for all five models. (a) Allelic model, (b) dominant model, and (c) recessive model.

**Figure 6:**
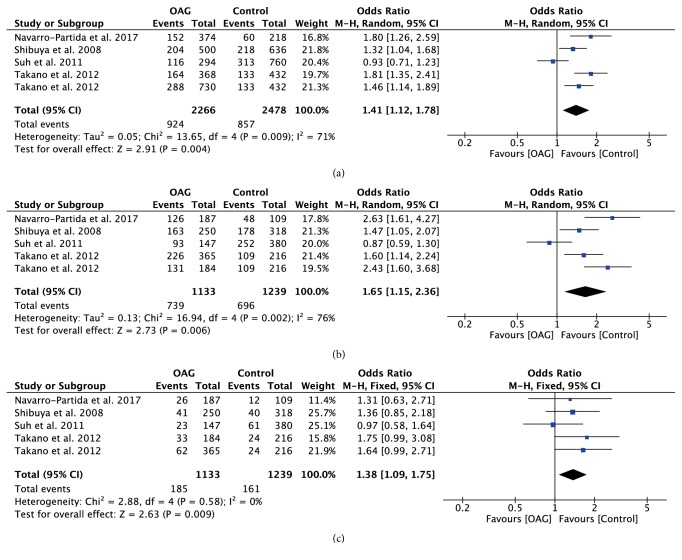
Forest plot of the association between TLR4 rs2149356 polymorphism and open angle glaucoma for all five models. (a) Allelic model, (b) dominant model, and (c) recessive model.

**Figure 7:**
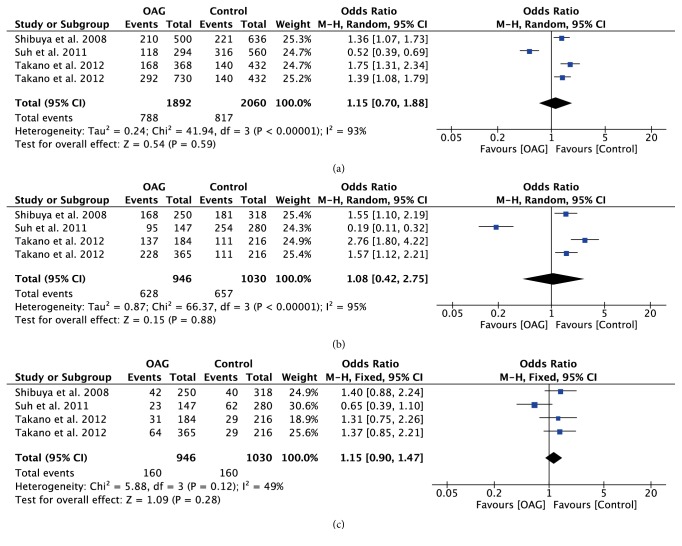
Forest plot of the association between TLR4 rs1927914 polymorphism and open angle glaucoma for all five models. (a) Allelic model, (b) dominant model, and (c) recessive model.

**Figure 8:**
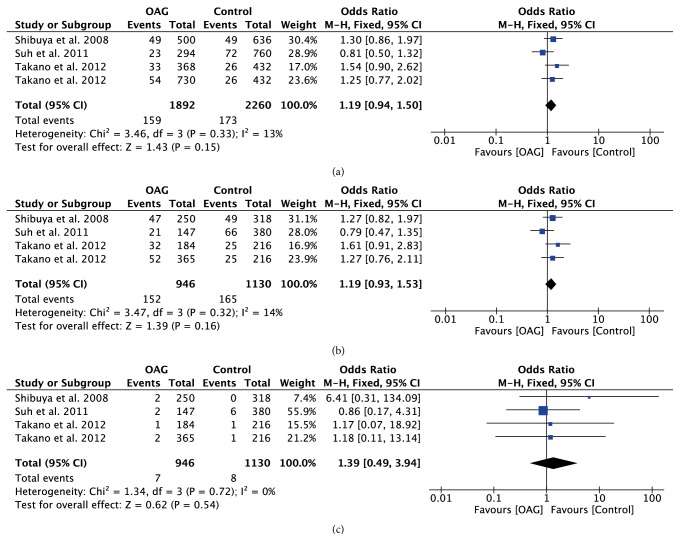
Forest plot of the association between TLR4 rs7045953 polymorphism and open angle glaucoma for all five models. (a) Allelic model, (b) dominant model, and (c) recessive model.

**Figure 9:**
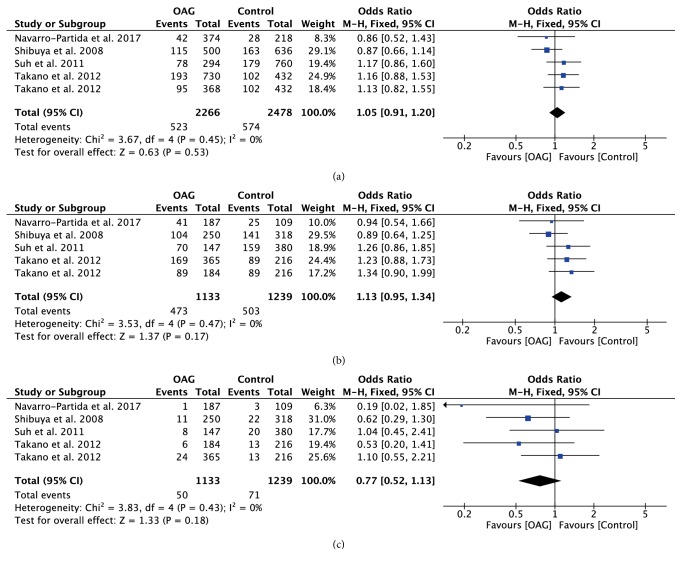
Forest plot of the association between TLR4 rs11536889 polymorphism and open angle glaucoma for all five models. (a) Allelic model, (b) dominant model, and (c) recessive model.

**Figure 10:**
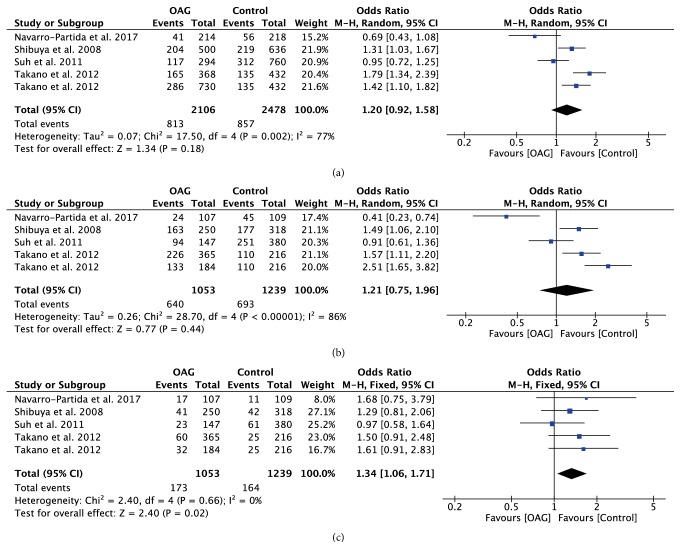
Forest plot of the association between TLR4 rs1927911 polymorphism and open angle glaucoma for all five models. (a) Allelic model, (b) dominant model, and (c) recessive model.

With regard to rs10759930, rs12377632, and rs2149356, the meta-analysis results showed significant differences in all genetic models (all P<0.05) (Figures 4–6), indicating that the mutant allele carriers had an increased frequency in OAG patients; thus we had reason to believe that these SNPs were associated with increased risk of OAG. However, there was significant heterogeneity among the available studies. When stratified by ethnicity, we detected that rs10759930 increased the OAG risk in Asians, while rs12377632 and rs2149356 increased the risk in both Asians and Mexicans. When stratified according to the subtype of glaucoma, significant associations were found in POAG patients rather than NTG patients, whereas, for rs1927914, rs7045953, and rs11536889, no significant associations were identified for any polymorphisms in overall analyses in any genetic model (all P>0.05) (Figures 7–9). Furthermore, the association of rs1927911 with OAG risk was found to be significant in recessive models (OR=1.34; 95%CI: 1.06-1.71; P=0.02) (Figure 10). Subgroup analysis indicated that the GG genotype might exert an increased risk in Asian POAG patients (Asian: OR=1.31; 95%CI: 1.02-1.69; P=0.04; POAG: OR=1.63; 95%CI: 1.03-2.59; P=0.04).

Funnel plots were not constructed to assess the publication bias due to relatively limited number of studies. Instead, Begg's test and Egger's test were adopted. The results of these genotype analyses showed no obvious publication bias (all P>0.05) (Table 3).

## 4. Discussion

Open-angle glaucoma is traditionally considered to be a multifactorial disease with strong evidence implicating a significant heritable component. Genetic linkage studies have so for identified more than 20 gene loci that contribute to the genetic risk of POAG, such as myocilin (MYOC), optineurin (OPTN), caveolins 1 and 2 (CAV1/CAV2), cyclin-dependent kinase inhibitor 2B antisense (CDKN2B), and growth arrest-specific 7 (GAS7) [4, 25]. TLRs are important contributors to the innate immune system and imbalance of both proapoptotic and protective regulation may lead to the degeneration of the RGCs in glaucoma, especially TLR4. Currently, studies about TLR4 polymorphisms in the ocular diseases were mainly focused on age-related macular degeneration (AMD), diabetic retinopathy (DR), and OAG. The role of TLR4 polymorphisms seemed to be negative and awaited further investigations in AMD patients [26–28], whereas some scholars associated TLR4 variants with susceptibility of DR [29–31]. However, studies concerning the association between TLR4 polymorphisms and OAG have presented contradictory conclusions.

In the present study, we undertook a systematic review and meta-analysis of associations between TLR4 polymorphisms and the presence of OAG, trying to figure out whether TLR4 SNPs could be regarded as a novel potential candidate genetic risk factor for OAG. TLR4 rs7037117, rs1927911, rs10759930, rs12377632, and rs2149356 might contribute to OAG susceptibility. Meta-analysis of the TLR4 rs1927914, rs11536889, and rs7045953 failed to reveal any significant associations with OAG. Since previous results of different studies have presented that ethnic differences may affect genetic predisposition to OAG [32–34], we also conducted subgroup analysis. When stratified by ethnicity, we detected that rs7037117, rs10759930, and rs1927911 might increase the risk in Asians, while rs12377632 and rs2149356 seemed to increase the risk in Asians and Mexicans. Chen et al. [35] recently conducted a systematic review and meta-analysis of all reported gene polymorphisms associated with POAG, and the results showed a significant association in 20 SNPs, which also included TLR4 polymorphisms. Their analyses showed that rs1927911 and rs2149356 in the TLR4 gene indicated significant association with POAG in Asians, which was consistent with our results. But our study emphasized and described in detail that ethnicity was one influence factor of TLR4 polymorphisms causing the risk of OAG. The enrolled studies were mainly carried out in East Asia, suggesting OAG is a complex disease with many associated factors, such as the environmental factors like dietary habits and lifestyle. However, whether the role of TLR4 polymorphisms in OAG is ethnic specific remains to be elucidated. More studies from different ethnic background, such as Caucasian and African, are needed to further define this association. When stratified by the subtype of glaucoma, our results demonstrated that TLR4 polymorphisms may have a tendency to influence the phenotypic features in POAG patients, instead of NTG patients, indicating that the pathogenesis and effect of TLR4 may be different between POAG and NTG. Chaiwiang and Poyomtip [36] conducted a similar meta-analysis recently and six allelic models were examined in their analysis. One notable difference was that their results indicated that only rs1927911 correlated with NTG in the homozygous model (GG vs. AA; OR=0.70; P=0.025), suggesting that individuals with the homozygous AA genotype exhibited increased risk of NTG when comparing with the GG homozygote. Further studies are required to provide supporting evidence. However, modest heterogeneity still existed in our estimates after we conducted stratification analyses. Thus, these results should be interpreted with caution.

TLR4 gene, located in chromosome 9q32-q33, participates in the activation of an inflammatory cascade through nuclear factor kappa-light-chain-enhancer of activated B cells (NF-*κ*B) [37]. The dysregulation of TLR4 signaling pathway during oxidative and ischemic injuries promotes axonal and neuronal loss. As is known to all, the mutations in 5'UTR can usually affect the expression of downstream gene by either translational regulation or impacting the cis-element binding of transcription factors. The sequence and structural features of the 3'UTR strongly influence the regulation of mRNA stability, translation, and localization. While the intronic SNPs do not have any direct influence on the conformation of the protein molecule, they are integral to gene expression regulation. Our meta-analysis results confirmed that certain mutations of some SNPs located on these sequences might increase the risk of OAG. Although the exact function of these TLR4 polymorphisms was not fully understood, there is a possibility that these SNPs might influence the mRNA stability and regulate the TLR4 gene expression, either the amount of TLR4 produced and/or relative levels of isoforms.

Polymorphisms of rs4986790 and rs4986791 in exon 3 are among the best-known and most often studied SNPs in TLR4 family. The genetic variation in the coding region of the TLR4 gene rs4986790 and rs4986791 could lead to variants in polypeptide chains; thus the coreceptor binding region would change and affect the signal transduction of the receptor in response to LPS stimulation. In other words, the missense mutation results in an amino acid substitution are associated with receptor hyporesponsiveness [38–40]. This might cause a dysfunction of the TLR4 molecule and interfere with the host immune system. Currently, two research teams from Saudi Arabia [18, 19] and Mexico [21] focused on rs4986790 and rs4986791 polymorphisms, only to yield inconsistent results. Confronting ethnic disparities, meta-analyses were not performed in these two SNPs. More studies should be performed in the future to assess these SNPs.

Nowadays, it is still not completely clear how TLR4 SNPs are associated with OAG, but a series of experimental evidence has addressed the role of TLR4 signaling in the pathophysiology of glaucoma. Luo et al. [13] demonstrated that TLR4 was upregulated in the retina of human glaucomatous eyes and the expression of TLR4 was prominently located on retinal microglia cells. TLR4 signaling was also identified in the regulation of the extracellular matrix in the trabecular meshwork [41, 42]. Morzaev et al. [10] revealed that TLR4 knockout mice had better preservation of RGCs after optic nerve crush, indicating that the TLR4-depedent pathway was involved in inducing the RGCs death. Furthermore, results from in vivo treatment experiments suggested that inhibition of TLR4-NF-*κ*B pathways would be an effective approach to prevent the apoptotic cascade of RGCs in glaucoma [11, 12]. Future studies should unravel how the studied SNPs functionally contribute to OAG.

The present study has some limitations. First, the number of studies included in the systematic review might have been relatively small, especially in the stratification analyses, making the results tentative, which await further confirmation in replication in more study cohorts. Second, heterogeneity is a potential problem that may affect the interpretation of the results, since heterogeneity obviously existed among studies for some polymorphisms, even after subgroup analyses according to ethnicity and subtypes for OAG. Third, only Asian and Mexican populations were involved in the final meta-analysis. Additional ethnic groups should be enrolled due to ethnic differences in gene polymorphisms. Finally, OAG is a multifactor disease related with genetic and environment factors. However, the potential roles of gene-gene and gene-environment interactions were not taken into consideration.

Nonetheless, our meta-analysis of case-control studies on TLR4 polymorphisms in OAG revealed that certain mutations of some TLR4 SNPs might increase the susceptibility of POAG in Asian and Mexican populations. However, TLR4 polymorphisms are still far from being candidate genetic biomarkers for OAG due to low statistical power and the presence of heterogeneity. The associations of TLR4 polymorphisms and their roles in the pathogenesis of OAG are still obscure and are not completely consistent in different ethnicities. More multicentric studies involving various ethnic groups and larger sample sizes are essential to provide a better ascertainment of the association between these TLR4 polymorphisms and OAG. Despite the tremendous progress in genes identified in glaucoma, further researches investigating gene polymorphisms are desiderated and expected to better understand this complex disease and help define disease specific targets, thus facilitating the development of diagnostic and therapeutic strategies.

## 5. Conclusion

The meta-analysis highlighted that certain mutations of some TLR4 polymorphisms might increase the susceptibility of OAG. However, TLR4 polymorphisms are still far from being candidate genetic biomarkers for OAG. Additional researches involving larger scale epidemiological studies are warranted to validate our results.

## Figures and Tables

**Table 1:** Characteristics of the studies included in the meta-analysis.

First author	Year	Diagnosis	Country	Cases			Control			TLR4 polymorphisms	NOS score
				No.	Age	F/M	No.	Age	F/M		
Shibuya et al.	2008	NTG	Japan	250	46.1 ± 7.7	131/119	318	61.2 ± 8.3	161/157	rs10759930, rs1927914, rs1927911, rs12377632, rs2149356, rs11536889, rs7037117, rs7045953	7
Suh et al.	2011	NTG	Korea	147	NA	NA	380	NA	NA	rs10759930, rs1927914, rs1927911, rs12377632, rs2149356, rs11536889, rs7037117, rs7045953	7
Takano et al.	2012	NTG	Japan	365	58.6 ± 13.1	194/171	216	69.7 ± 11.3	100/116	rs10759930, rs1927914, rs1927911, rs12377632, rs2149356, rs11536889, rs7037117, rs7045953	7
		POAG		184	64.6 ± 14.3	65/119	216	69.7 ± 11.3	100/116		
Chen et al.	2012	POAG	China (Hong Kong)	184	59.7 ± 16.6	64/120	230	73.5 ± 7.5	124/106	rs7037117	7
			China (Shantou)	102	45.2 ± 20.8	20/82	147	74.0 ± 6.4	94/53		
			China (Beijing)	176	38.9 ± 16.3	38/138	200	69.4 ± 6.0	50/150		
Mousa et al.	2016	POAG	Saudi Arabia	85	60.9 ± 12.7	32/53	95	58.7 ± 10.4	38/57	rs4986791	8
Abu-Amero et al.	2017	POAG	Saudi Arabia	85	60.9 ± 12.7	32/53	95	58.7 ± 10.4	38/57	rs4986790	8
Navarro-Partida et al.	2017	POAG	Mexico	187	66.49 ± 14.3	95/94	109	63.28 ± 7.93	69/40	rs4986790, rs4986791	8
Navarro-Partida et al.	2017	POAG	Mexico	187	66.49 ± 14.3	95/94	109	63.28 ± 7.93	69/40	rs1927911, rs12377632, rs2149356, rs11536889	8

NTG= normal tension glaucoma, POAG= primary open angle glaucoma, No.=number, F/M=female/male, and NOS= Newcastle-Ottawa Scale; NA=not applicable.

**Table 2:** Meta-analysis of the association between TLR4 polymorphisms and OAG.

SNP	Variables	No. of studies	Allelic model (2 vs. 1)			Dominant model (22+12 vs. 11)			Recessive model (22 vs. 12+11)		
			OR (95%CI)	P	I^2^ (%)	OR (95%CI)	P	I^2^ (%)	OR (95%CI)	P	I^2^ (%)
rs7037117*∗*	Total	5	1.25 (1.09,1.44)	0.002	46	1.26 (0.94,1.69)	0.12	66	1.49 (1.08,2.04)	0.01	4
Diagnosis	POAG	2	1.19 (0.83,1.70)	0.34	53	1.15 (0.60,2.19)	0.68	80	1.71 (1.10,2.66)	0.02	40
	NTG	3	1.27 (0.97,1.68)	0.09	61	1.34 (0.93,1.92)	0.11	66	1.28 (0.82,2.01)	0.28	0
rs10759930*∗*	Total	4	1.31 (1.04,1.65)	0.02	68	1.50 (1.04,2.19)	0.03	75	1.29 (1.01,1.66)	0.04	0
Diagnosis	POAG	1	1.71 (1.29,2.29)	0.0002	NA	2.51 (1.65,3.83)	<0.0001	NA	1.41 (0.82,2.45)	0.22	NA
	NTG	3	1.21 (0.96,1.53)	0.11	61	1.29 (0.94,1.78)	0.11	58	1.26 (0.95,1.67)	0.10	0
rs1927914*∗*	Total	4	1.15 (0.70,1.88)	0.59	93	1.08 (0.42,2.75)	0.88	95	1.15 (0.90,1.47)	0.28	49
Diagnosis	POAG	1	1.75 (1.31,2.34)	0.0001	NA	2.76 (1.80,4.22)	<0.0001	NA	1.31 (0.75,2.26)	0.34	NA
	NTG	3	1.00 (0.55,1.82)	0.99	94	0.78 (0.25,2.49)	0.68	96	1.09 (0.68,1.76)	0.71	65
rs7045953*∗*	Total	4	1.19 (0.94,1.50)	0.15	13	1.19 (0.93,1.53)	0.16	14	1.39 (0.49,3.94)	0.54	0
Diagnosis	POAG	1	1.54 (0.90,2.62)	0.11	NA	1.61 (0.91,2.83)	0.10	NA	1.17 (0.07,18.92)	0.91	NA
	NTG	3	1.12 (0.86,1.45)	0.41	15	1.11 (0.84,1.47)	0.47	7	1.43 (0.46,4.39)	0.54	0
rs1927911	Total	5	1.20 (0.92,1.58)	0.18	77	1.21 (0.75,1.96)	0.44	86	1.34 (1.06,1.71)	0.02	0
Ethnicity	Asian	4	1.33 (1.04,1.69)	0.02	70	1.52 (1.05,2.20)	0.03	75	1.31 (1.02,1.69)	0.04	0
	Mexican	1	0.69 (0.43,1.08)	0.10	NA	0.41 (0.23,0.74)	0.003	NA	1.68 (0.75,3.79)	0.21	NA
Diagnosis	POAG	2	1.13 (0.44,2.88)	0.80	92	1.03 (0.17,6.07)	0.97	96	1.63 (1.03,2.59)	0.04	0
	NTG	3	1.22 (0.97,1.53)	0.10	59	1.31 (0.95,1.80)	0.10	58	1.25 (0.94,1.66)	0.12	0
rs12377632	Total	5	1.33 (1.06,1.67)	0.01	70	1.53 (1.11,2.09)	0.009	67	1.32 (1.05,1.66)	0.02	46
Ethnicity	Asian	4	1.30 (0.99,1.71)	0.06	76	1.46 (1.02,2.10)	0.04	74	1.27 (0.86,1.88)	0.23	57
	Mexican	1	1.50 (1.07,2.10)	0.02	NA	1.95 (1.07,3.54)	0.03	NA	1.54 (0.92,2.57)	0.10	NA
Diagnosis	POAG	2	1.71 (1.37,2.14)	<0.001	3	2.18 (1.55,3.06)	<0.001	0	1.82 (1.25,2.63)	0.002	0
	NTG	3	1.16 (0.93,1.44)	0.18	54	1.28 (0.90,1.81)	0.17	65	1.08 (0.81,1.45)	0.59	0
rs2149356	Total	5	1.41 (1.12,1.78)	0.004	71	1.65 (1.15,2.36)	0.006	76	1.38 (1.09,1.75)	0.009	0
Ethnicity	Asian	4	1.34 (1.04,1.73)	0.02	74	1.49 (1.02,2.18)	0.04	76	1.39 (1.08,1.79)	0.01	0
	Mexican	1	1.80 (1.26,2.59)	0.001	NA	2.63 (1.61,4.27)	<0.001	NA	1.31 (0.63,2.71)	0.47	NA
Diagnosis	POAG	2	1.81 (1.44,2.26)	<0.001	0	2.51 (1.83,3.44)	<0.001	0	1.56 (1.00,2.45)	0.05	0
	NTG	3	1.22 (0.94,1.59)	0.13	67	1.29 (0.91,1.83)	0.16	65	1.31 (0.99,1.74)	0.06	2
rs11536889	Total	5	1.05 (0.91,1.20)	0.53	0	1.13 (0.95,1.34)	0.17	0	0.77 (0.52,1.13)	0.18	0
Ethnicity	Asian	4	1.06 (0.92,1.23)	0.41	1	1.15 (0.96,1.37)	0.13	3	0.81 (0.55,1.20)	0.29	0
	Mexican	1	0.86 (0.52,1.43)	0.56	NA	0.94 (0.54,1.66)	0.84	NA	0.19 (0.02,1.85)	0.15	NA
Diagnosis	POAG	2	1.04 (0.79,1.37)	0.76	0	1.19 (0.86,1.65)	0.29	0	0.44 (0.18,1.09)	0.08	0
	NTG	3	1.05 (0.89,1.23)	0.58	31	1.10 (0.90,1.35)	0.34	16	0.88 (0.57,1.36)	0.57	0

OR: odds ratio; 95%CI: 95% confidence interval; POAG: primary open angle glaucoma; NTG: normal tension glaucoma; NA: not applicable.

*∗*The involved studies for these polymorphisms only included Asian populations; thus the overall results also represented the results in Asian subgroup.

**Table 3:** Begg's and Egger's test for publication bias in the meta-analysis of the association between TLR4 polymorphisms and OAG.

SNP	Alleles (1/2)	Allelic model		dominant model		recessive model	
		(2 vs. 1)		(22+12 vs. 11)		(22 vs. 12+11)	
		P (Begg)	P (Egger)	P (Begg)	P (Egger)	P (Begg)	P (Egger)
rs7037117	A/G	0.462	0.567	0.221	0.372	0.368	0.233
rs10759930	T/C	1	0.869	0.734	0.648	0.734	0.642
rs1927914	A/G	0.734	0.734	0.734	0.658	0.308	0.481
rs7045953	A/G	1	0.936	1	0.925	0.089	0.335
rs1927911	G/A	1	0.443	0.462	0.529	0.462	0.428
rs12377632	C/T	0.806	0.576	0.806	0.597	1	0.873
rs2149356	G/T	0.462	0.400	0.462	0.285	1	0.991
rs11536889	G/C	0.806	0.698	0.462	0.923	0.806	0.323

## Data Availability

All data are fully available in the paper without restriction.
